# Variable practice is superior to self-directed training for laparoscopic simulator training: a randomized trial

**DOI:** 10.1007/s00464-024-10688-z

**Published:** 2024-02-06

**Authors:** Anishan Vamadevan, Lars Konge, Flemming Bjerrum

**Affiliations:** 1 Copenhagen Academy for Medical Education and Simulation, University of Copenhagen and Centre for HR and Education, Capital Region, Ryesgade, 53B, 2100 Copenhagen, Denmark; 2 Faculty of Health and Medical Sciences, University of Copenhagen, Copenhagen, Denmark; 3 Surgical Section, Copenhagen University Hospital, Amager and Hvidovre, Hvidovre, Denmark

**Keywords:** Variable practice, Laparoscopy, Simulation, Retention, Skills acquisition, Feedback, Transfer, Cognitive load, Proficiency

## Abstract

**Background:**

Mastering laparoscopy is challenging—it requires specific psychomotor skills which are difficult to obtain in the operating room without potentially compromising patient safety. Proficiency-based training programs using virtual reality simulators allow novices to practice and develop their skills in a patient-safe learning environment. Variable practice leads to stronger retention and skills transfer in a non-surgical setting. The objective of this trial was to investigate if variable practice was superior to self-directed training.

**Methods:**

A randomized trial where participants (*n* = 36) were randomized to proficiency-based laparoscopic simulator training of basic skills using either variable practice or self-directed training, followed by a transfer test with proficiency-based training on a procedural task (a salpingectomy). All participants returned after a period of 3–5 weeks to perform a retention test. Results: The mean time to proficiency for the basic skills tasks were 119 min (SD: 93) for the variable practice group versus 182 min (SD: 46) for the self-directed training group (*p* = 0.015). The time to reach proficiency during the transfer test was 103 min (SD: 57) versus 183 min (SD: 64) for the variable practice group versus the self-directed training group, respectively (*p* < 0.001). The mean time to proficiency for the retention test was 51 min (SD: 26) and 109 min (SD: 53) for the variable practice group and self-directed training group, respectively (*p* < 0.001).

**Conclusion:**

Variable practice is superior to self-directed training for proficiency-based laparoscopic training. With variable time to practice proficiency is reduced, there is higher transfer to a procedural task, and retention is improved.

**Supplementary Information:**

The online version contains supplementary material available at 10.1007/s00464-024-10688-z.

Simulation-based skills training is a standard part of modern surgical training and allows novice surgeons to practice without the risk of compromising patient safety [1, 2]. Studies show that skills obtained in a simulated setting are transferable to the operating room leading to a reduction in operating time and a reduced risk of mistakes during initial operations [3, 4]. Virtual reality simulators are effective training tools and can help ensure proficiency; however, how training is organized is essential for optimal training outcomes [5, 6]. Therefore, it is essential to identify the best training strategies that lead to optimal long-term consolidation of skills [7].

Variable practice is a training approach where tasks are practiced at random rather than in a pre-specified order, e.g., increasing degree of difficulty or in a self-directed manner.

Variable practice has, in educational literature, been suggested as an alternative to the traditional scaffolding approach, where trainees complete one task at a time. The benefits of variable practice were first hypothesized and explored by Battig et al. [8, 9], who argued that the contextual interference effect caused by an increase in variability of tasks during skills training leads to an initial impairment of the performance, but to stronger long-term retention and increased adaptability. Increasing variability of tasks also increases the mental demand of the trainees who constantly must adapt to the new randomly assigned tasks. Variable practice has since been explored in non-surgical motor learning studies and has demonstrated increased transfer effect and retention of skills [10–12]. In contrast, previous studies in surgical skills acquisition on variable practice versus blocked training have found mixed results and failed to demonstrate the superiority of variable practice [13–16]. A possible explanation for this could be that prior studies only allowed participants to practice for a limited amount of time or a certain number of repetitions which only examines the effect on the initial part of the trainee’s learning curve. The impact of variable practice on proficiency-based training has yet to be explored.

Instructor-based feedback reduces the time to reach proficiency in a laparoscopy training program without negatively impacting the retention of skills [17, 18]. However, having instructors available is an additional cost to simulation-based training, and using variable practice instead of self-directed training could increase the need for instructor-based feedback since variable practice could be more challenging [19].

The objective of this trial was to examine if training using a variable practice approach was superior to a self-directed approach in terms of retention and transfer to a procedural task. We also explored how variable practice affected the need for instructor feedback and the mental and cognitive load experienced during training.

## Materials and method

A single-center randomized superiority trial was planned according to the extended CONSORT statement for Health Care Simulation-Based Research [20]. The Regional Committee on Biomedical Research Ethics exempted the trial from ethical approval (F-22051506). The trial was registered at Clinicaltrials.gov (NCT05731674).

### Setting

Data were collected at the Copenhagen Academy for Medical Education and Simulation (CAMES) simulation center in Copenhagen, Denmark [21]. All data were collected by the principal investigator.

### Participants

Participants were medical students—novices without experience in laparoscopy. The inclusion and exclusion criteria used for the trial were as follows:

*Inclusion criteria for both intervention and follow-up:* (1) Medical students enrolled at a Danish university; (2) Provided informed consent before inclusion.

*Exclusion criteria for intervention*: (1) Having previously participated in studies involving laparoscopic training; (2) Having participated in laparoscopic training programs at any simulation center; (3) Having prior experience with laparoscopic surgery (having performed any laparoscopic procedures as a primary surgeon, including supervised procedures); (4) Having performed any supervised laparoscopy procedure as primary surgeons during the entire trial; (5) Did not give consent; (6) Did not speak Danish on a conversational level.

All participants received written and verbal information before signing a written informed consent form. All were given a unique trial identification number before randomization.

Medical students were recruited using advertisements in their student newspaper and the social media platform Facebook using a group-specific for medical students enrolled at the University of Copenhagen. Students proved their eligibility by documenting their enrollment.

### Intervention

Before randomization, participants would receive a short introduction to the simulator and the instruments, how to adjust the ergonomic aspects, and how to access and read the simulator’s instructions and watch the instructional videos for the basic skills. Participants were allowed to read the simulator’s instruction guides and watch the instruction videos as often as they wanted.

All participants had proficiency-based training of basic skills but were randomized to using either a variable practice approach (intervention group) or a self-directed approach (control group) on a virtual reality laparoscopy simulator. Both groups practiced the same four basic skills tasks (Cutting, Grasping, Lifting and grasping, and Fine dissection) to proficiency.

**Fig. 1:**
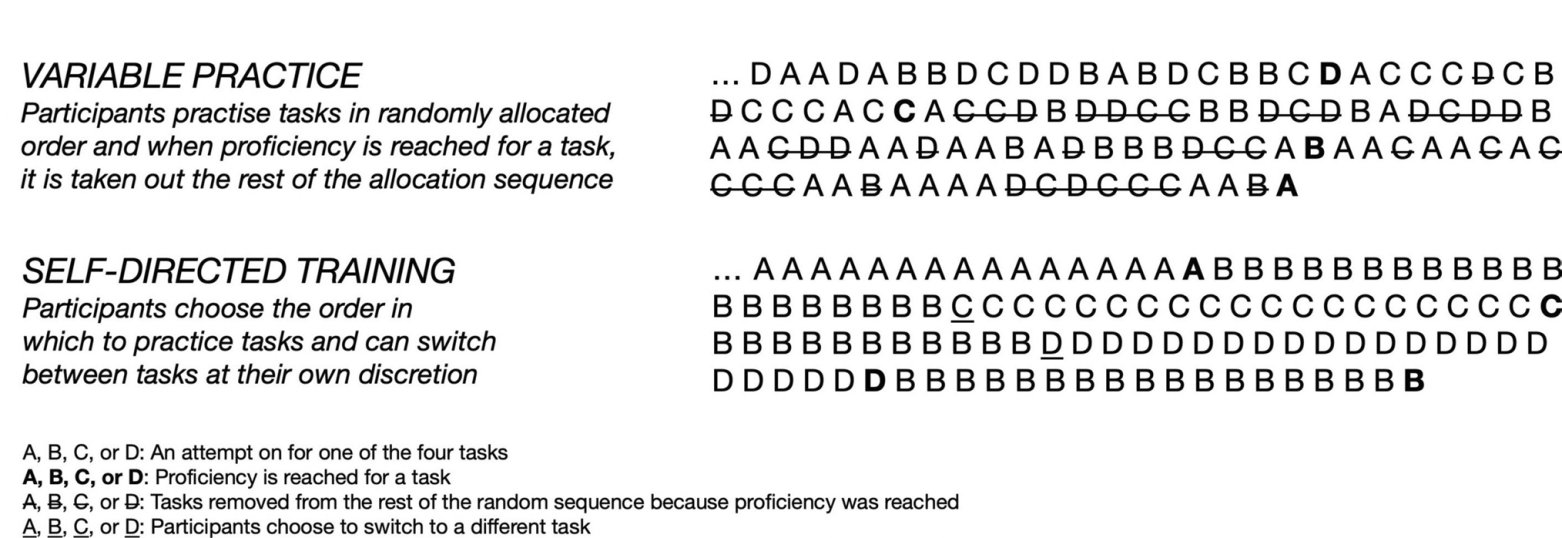
An example of task sequence during training using the variable practice group (intervention) and self-directed training group (control) respectively. Each letter represents as attempt at one of the four basic skills tasks (A, B, C, or D) used in the interventions

Participants in the variable practice group would receive a unique string of tasks evenly distributed between the four basic skills in random order. The tasks they should practice were displayed on a monitor beside the simulator. Furthermore, the string was blinded, meaning that the trainee could only see the task at hand and could only see the next one once the prior task was completed, regardless of the outcome. Once proficiency for a specific task was reached twice within five consecutive attempts for that task specifically, it was removed from the string. This would prevent overtraining as this could be a potential bias for the retention test, which the variable practice group could benefit from. The self-directed group could practice the basic skills tasks in the order they preferred and were allowed to alternate between the tasks as they pleased, Fig. 1. Participants from the self-directed group also had to pass all four basic skills tasks twice within five consecutive attempts. Once a task was passed twice, participants from the self-directed group were also not allowed to practice that specific task any further; this was also to prevent overtraining.

Having to pass twice within five consecutive attempts minimized the chance of passing by chance. However, the two passing attempts did not have to be consecutive due to the difficulty of the tasks. The proficiency levels used as training goals have been used in previously published trials. They included parameters such as instrument time, instrument path length, instrument angular path length, tissue damage, bleeding, energy damage, etc., which all had to be passed simultaneously [18, 22] (Supplementary Materials). The simulator would automatically assess all parameters after every attempt, to determine if the trainee had passed or failed the specific attempt.

### Transfer test

After completing the four Basic Skills tasks, both groups performed a transfer test. A transfer test can determine whether newly acquired skills, using one training approach, result in improved performance in a new setting or on a different task compared to another approach [23].

We chose proficiency-based training of procedural task, a salpingectomy with bleeding due to an ectopic pregnancy, as the transfer test. Trainees had access to an instructional video on how to perform the salpingectomy, as well as written instructions. The proficiency levels used as training goals have been used in previously published trials and were based on parameters such as instrument time, instrument path length, instrument angular path length, tissue damage, bleeding, energy damage on blood vessels or an ovary, etc., which all had to be passed simultaneously[24] (Supplementary Materials). To complete the transfer test, trainees had to pass twice within a maximum of five consecutive attempts. We chose the procedural module for a salpingectomy with bleeding because prior studies have shown this module to be difficult and because it resembles an actual clinical procedure [22, 25]. By using a more difficult proficiency-based test, we also reduced the risk of a “ceiling effect” that would make it difficult to distinguish any potential difference in transfer.

To prevent the influence of simulation fatigue and to make a clear cut-off point from the intervention, trainees were not allowed to start the transfer test on the day they had completed all the Basic Skills tasks. Instead, they were invited back, the earliest being the following day.

### Retention test

To examine any difference in the retention of skills, all participants had to return after a retention period of 3–5 weeks, where no simulation-based training nor performing surgery as the lead surgeon was allowed. During the retention test, both groups had to practice until proficiency again using the self-directed approach on the simulator for all five tasks. We chose the self-directed approach for the retention test because this is currently considered the gold standard for simulation-based training [26, 27]. Participants had to reach proficiency for all tasks, both the basic skills and the procedural task, for the retention test, which entails passing all tasks twice within a maximum of five consecutive attempts.

During the entire trial, participants booked training sessions by e-mail, and a maximum of one session consisting of a maximum of 2-h was allowed per day to minimize cognitive overload and fatigue.

During the entire trial, instructor assistance was given upon request, and the principal investigator recorded the duration of the interaction.

### Randomization

A 1:1 randomization was performed centrally using a web-based system from Sealed Envelope® (London, United Kingdom). The allocation sequence was computer-generated and used varying block sizes of four and six, a sequence that was kept concealed from the principal investigator throughout the trial. Participants were stratified according to sex (men/women) [28]. Blinding was not possible due to the nature of the study.

### Materials and equipment

Three identical non-haptic LapSim® virtual reality simulators (software version 2016.1) from Surgical Science (Gothenburg, Sweden) connected to a common server were used for this trial.

All simulators were connected to a 27” monitor and were height adjustable to ensure the best viewing condition and the most ergonomically correct working position. If multiple participants were training during the same time slot, they would be required to wear noise-canceling Bose Quiet Comfort III headsets (Bose Corp., Massachusetts, USA).

### Outcomes

The primary outcome was the total time (minutes) spent to reach the predefined proficiency level for all five tasks during the retention test.

The secondary outcome was the total time (minutes) to reach the predefined proficiency on the procedural task and the objective was to investigate transfer to a more complex task.

Exploratory outcomes were time to reach proficiency for the basic skills during the intervention and the time (seconds) where participants needed instructor feedback during training and the cognitive workload using the SIM-TLX questionnaire. To assess the mental and cognitive load experienced during training, we used the SIM-Task Load Index (SIM-TLX) questionnaire [29]. The SIM-TLX is a validated questionnaire used to assess the perceived cognitive load for simulation training consisting of nine dimensions: mental demands, physical demands, temporal demands, frustration, task complexity, situational stress, distractions, perceptual strain, and task control. It uses a numerical scale from 0–100 for each dimension. The SIM-TLX questionnaire was filled out by all participants after the first and final session of the intervention.

### Sample size calculation

The sample size was calculated for the primary outcome (time to reach proficiency for the retention test) based on data from a previous trial [22]. For the control group, using the self-directed approach, it was assumed that a mean time of 95 min was needed to reach proficiency. A minimum reduction of 25% was estimated to be relevant, so the meantime for the intervention group was set to 71 min. The standard deviation was set to 18 for both groups. Using a two-sided significance level of 0.05 and the power set at 0.95, the minimum sample size required was 30 participants, a minimum of 15 in each group.

### Statistical analysis

The data were analyzed using SPSS® 28.0 (IBM, Armonk, NY, USA). Independent *t*-tests were used for intergroup comparisons of the primary, secondary, and exploratory outcomes. Mann–Whitney *U* tests were performed for non-parametric outcomes in relation to the exploratory outcomes.

Retention was defined as a reduction in training time to reach proficiency for both the basic skills tasks and the procedural task. To analyze the training effect, over time, for both the intervention and the control group, the mixed model with repeated measurements and an unstructured covariance matrix model was used for time to reach proficiency. The basic model was *Y* = *a* + *bI* + *ct* + *dt I*, where *I* is the indication of the intervention, *t* is time (time1 corresponding to the intervention and transfer phase and time2 corresponding to the retention phase (including both basic skills tasks and procedural task)), and *a* through *d* are coefficients of the regression equation.

## Results

**Fig. 2:**
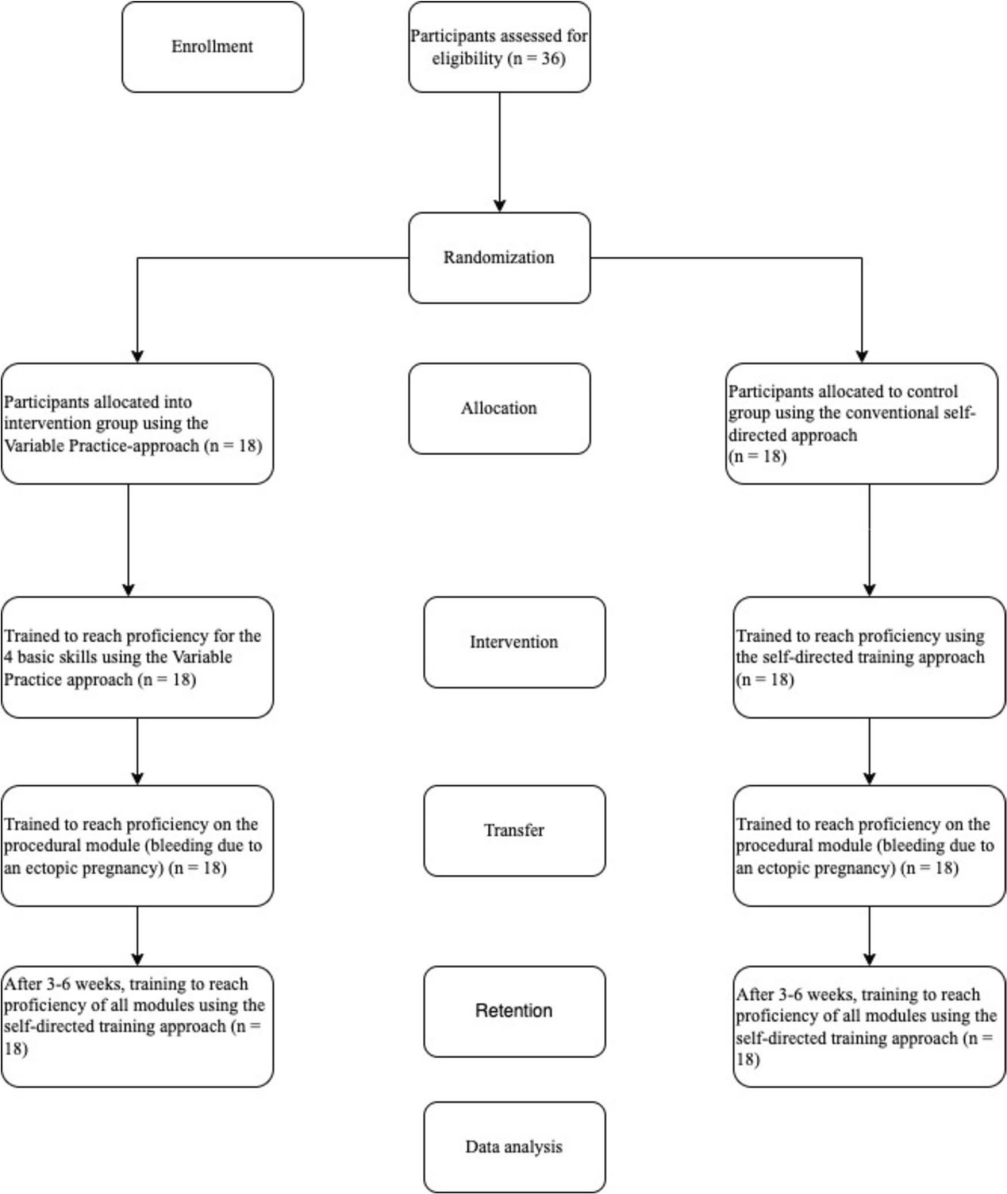
Flowchart according to the CONSORT Statement [20]

Thirty-six participants were included and randomized; all 36 completed both the intervention, the transfer, and the retention test (Fig. 2). Participant demographics are presented in Table 1.

**Table 1:** Baseline characteristics for participants who completed the intervention

Groups	Variable practice group	Self-directed practice group
Sex (number of men/women)	10/8	11/7
Age (median/range)	22 (19–33)	22 (20–29)
Dexterity (number of right-/left-handed	17/1	15/3

**Fig. 3:**
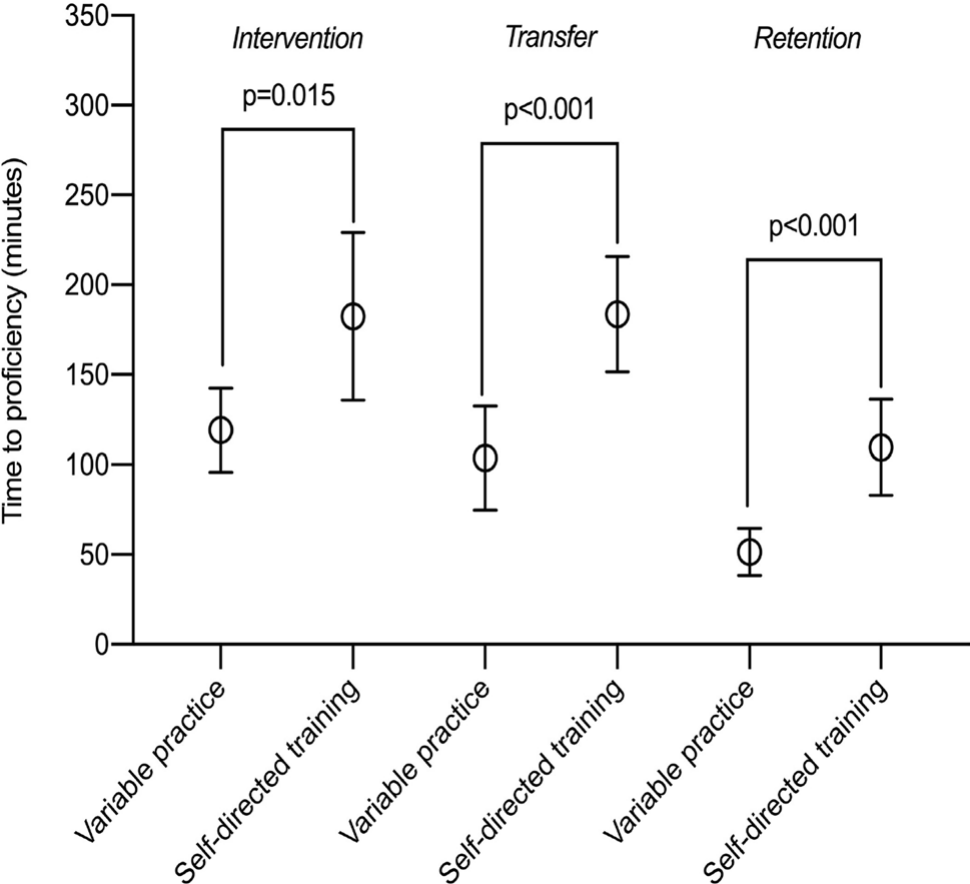
Interval plot of time to reach proficiency for the variable practice group and the self-directed training group during the intervention, the transfer test, and the retention phase

The variable practice group reached proficiency significantly faster for the basic skills during the intervention phase (*p* = 0.015) compared with the self-directed training group, Fig. 3. We found that the variable practice group reached proficiency significantly faster during the transfer test on the procedural task, as well (*p* < 0.001), Fig. 3. After the retention period of 3–5 weeks, the variable practice group achieved proficiency significantly faster overall (*p* < 0.001), but also for the basic skills (*p* = 0.026) and the procedural task (*p* < 0.001) alone, compared to the self-directed group, Fig. 3 and Table 2. Both the variable practice group (*p* < 0.001) and the self-directed group (*p* < 0.001) reduced the time to reach proficiency significantly during the retention test compared with the intervention for both the basic skills tasks and the procedural task, Table 2.

**Table 2:** Time to reach proficiency (All values reported as mean values and 95% CI)

		Variable practice group (*n* = 18)	Self-directed practice group (*n* = 18)	*P-*values
Intervention phase	Time to reach proficiency on basic skills tasks (minutes)	119 [96;143]	182 [136;229]	0.015
Transfer test	Time to proficiency on procedural task (minutes)	104 [75;133]	183 [151;216]	< 0.001
	Total time to reach proficiency for the four basic skills tasks and the procedural task (minutes)	223 [184;262]	366 [300;432]	< 0.001
Retention phase	Time to reach proficiency on Basic skills tasks (minutes)	27 [20;33]	48 [30;66]	0.026
	Time to proficiency on procedural task (minutes)	25 [17;33]	62 [45;78]	< 0.001
	Total time to reach proficiency for the four basic skills tasks and the procedural task (minutes)	51 [38;65]	110 [83;136]	< 0.001

**Fig. 4:**
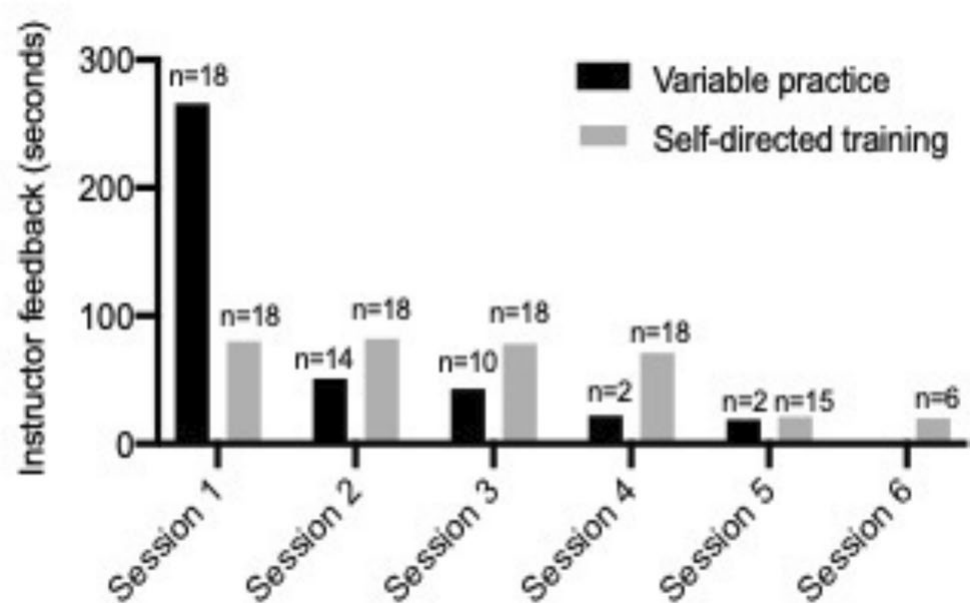
Bar chart showing the mean time (second) of instructor feedback during each training session during the intervention. The number of participants who requested feedback is listed above each bar

There were no statistically significant differences in the amount of assistance needed from the instructor during both the intervention (*p* = 0.77) and the retention phase (*p* = 0.74), Table 3. There was a significant difference in the amount of assistance needed from the instructor during the transfer test; the variable practice group needed significantly less instructor time compared to the self-directed training group (*p* < 0.001), Table 3. There was no significant difference in the number of times a participant from either group requested feedback during the intervention (*p* = 0.56), Table 3. However, there was a significant difference in the number of practice sessions where they needed instructor feedback during the intervention (*p* = 0.002). Furthermore, the variable practice group required more feedback during the beginning of training, whereas the self-directed training group required feedback distributed over all the training sessions, Fig. 4.

**Table 3:** Data on duration and frequency of instructor-based feedback

	Variable practice group (*n* = 18)	Self-directed practice group (*n* = 18)	*P-*values
Intervention, Basic skills task (seconds)^a^	388 [341;435]	397 [348;447]	0.77
Intervention, Procedural task (seconds)^a^	307 [263;351]	425 [378;473]	< 0.001
After 3–5 weeks of retention, basic skills and procedural tasks (seconds)^a^	44 [25;63]	48 [31;64]	0.74
Total number of times requesting feedback during intervention^b^	7 (7–8)	7 (7–8)	0.71
Number of training sessions where feedback was requested during intervention^b^	3 (2–2)	5 (5–6)	0.004

^a^Values are reported as mean values and 95% CI

^b^Values are reported as median and interquartile range (IQR)

After the first practice session during the intervention phase, we found that the variable practice group experienced a significantly higher amount of mental demand (*p* < 0.001), frustration (*p* < 0.001), and perceived task complexity (*p* < 0.001), Table 4. For the last training session during the intervention, the SIM-TLX revealed that the self-directed group perceived a higher amount of task complexity compared to the intervention group (*p* < 0.001).

**Table 4:** Comparison of subjective workload scores for novices using the SIM-TLX (Simulation Task Load Index)

	Workload after 1st Session			Workload after final session		
	Variable practice group (*n* = 18)	Self-directed practice group (*n* = 18)	*P*-value	Variable practice group (*n* = 18)	Self-directed practice group (*n* = 18)	*P*-value
Mental Demands	75 (7)	58 (9)	< 0.001	52 (8)	51 (8)	0.58
Physical Demands	40 (7)	41 (6)	0.44	33 (7)	32 (7)	0.72
Temporal Demands	44 (11)	37 (13)	0.14	45 (8)	42 (7)	0.271
Frustration	81 (12)	60 (12)	< 0.001	51 (9)	51 (9)	0.64
Task Complexity	79 (11)	57 (13)	< 0.001	50 (7)	73 (8)	< 0.001
Situational Stress	66 (6)	67 (8)	0.57	58 (6)	59 (5)	0.36
Distractions	16 (4)	14 (3)	0.077	16 (3)	16 (2)	0.56
Perceptual strain	50 (6)	49 (7)	0.62	37 (8)	35 (7)	0.39
Task control	55 (5)	56 (6)	0.66	41 (4)	40 (5)	0.44

Values are reported as means and standard deviation

## Discussion

We found that variable practice is superior to conventional self-directed training for a proficiency-based laparoscopy simulator training program. Variable practice reduced the time to reach proficiency, resulted in higher transfer to a procedural task, and improved retention due to a stronger consolidation of skills compared with self-directed training. Both groups significantly reduced their time to reach proficiency during the retention test compared to during the intervention. The reduction in time to reach proficiency demonstrates that this proficiency-based training program is resistant to skills decay over a period of 3–5 weeks. Similar results have been demonstrated in prior studies conducted by our research group using the same research methodology [17, 19, 22, 25]. Furthermore, our study demonstrates that the intervention group, who used a variable practice approach, is more resistant to the decay of skills than the control group. This is probably due to a stronger consolidation of skills for the variable practice group during the intervention.

Previous studies have examined the effect of variable practice for laparoscopic training. Still, none have found it superior to either blocked- or self-regulated training regarding retention of skills, skills transfer, or acquisition of skills in general [13–16].

Our study is the first trial to examine the effect of variable practice in the context of proficiency-based training, which is the gold standard for modern simulation training. Using proficiency-based training is a strength of our trial, in contrast to using time or repetition-limited training, where the individual learning curve is not considered. Previous studies have used time- or repetition-based training, where the individual learning curve is not considered [13–16]. It is possible that using a limited amount of training time or a fixed amount of practice repetitions limits the effect of the intervention. In easier tasks where proficiency is achieved early within the first few repetitions, participants would be forced to practice further, resulting in overtraining. This could potentially dilute the effect of the intervention for the following transfer- and retention test. Using proficiency-based criteria as a clean cut-off point makes the groups more comparable. In our study, we chose to use the time to proficiency for the procedural task as a transfer test [22]. In previous studies which used a transfer test after the intervention participants were not allowed to practice till proficiency, which could be a reason for their inconclusive results in regard to the transfer of skills as the transfer test was too short or not difficult enough [13, 14, 16].

Battig et al. argued that using a high variability of training would result in an initial impairment but an increased transfer and higher retention of skills [8, 9]. We found both higher retention and transfer to a more difficult task, but surprisingly we observed that participants in the variable practice group completed the basic skills faster than the control group. Although the variable practice group was faster overall, they needed more instructor feedback initially. An explanation for why the variable practice achieved proficiency significantly faster during the intervention could be that there was a transfer of skills between the four basic skills tasks. By forcing participants to practice different tasks randomly, participants were earlier exposed to different aspects of instrument handling and different viewing conditions. These variations could be beneficial to a better and earlier understanding of how to optimally manipulate the instruments and contribute to the trainee’s spatial orientation. These findings align with other studies outside of laparoscopic training, which argue that early and multiple exposures to different scenarios enhance the overall learning of the trainee [30].

Although there was no significant difference in the overall amount of instructor feedback time or the number of times feedback was requested during the intervention, we interestingly found that the use of feedback was used differently as the variable practice group used it most during the initial part of their training, whereas the control groups requested it throughout the entire intervention phase. The earlier exposure to all the tasks created an increased need for feedback during the beginning of the training for the variable practice group but less at the end of the intervention. This did not negatively impact their acquisition of skills; the variable practice group managed to complete the training using the same amount of feedback, but massed at the beginning of their training, and still complete the training program significantly faster than the self-directed group.

However, the variable practice requested significantly less instructor feedback when practicing the procedural task. So not only did the variable practice demonstrate a higher transfer by achieving proficiency for the procedural task faster, but they also did so more independently. There was no significant difference in the need for instructor feedback during the retention test. This indicates that the participants had obtained the same knowledge on how to solve the four basic skills and the procedural task during the intervention. However, the skills they had acquired were different in terms of quality, as the variable practice group completed the retention test significantly faster. This is consistent with the findings by Lee and Carnahan [31].

According to cognitive load theory, learning is most effective when learners have enough mental capacity to reflect on the task they are learning (germane load) in addition to the resources required to complete the task (intrinsic load) and understand the instructions and filter out distractions (exogenous load) [32–34]. The intrinsic load increases by increasing variability and thereby increasing the difficulty of training for trainees. Therefore, the initial training performance could be impaired due to an excessive cognitive load and become less effective by using a varied practice approach. This would favor blocked training for short-term skills acquisition as the intrinsic load is lower. The SIM-TLX questionnaire revealed that the variable practice group perceived the training during the first session of the intervention to be significantly more mentally challenging (Mental Demand), experienced more frustration (Frustration), and perceived the training session to be more complex (Task Complexity) compared to the self-directed training group. The increased need for instructor feedback and the higher cognitive load at the beginning of training indicate that there was, possibly, an initial impairment of skills during the first session due to the high variability of training. The increased need for instructor feedback and the initial increased cognitive load experienced by the variable practice group was probably due to earlier exposure to both more different tasks as well as more challenging tasks.

During the last session of the intervention, the SIM-TLX questionnaire revealed no differences in perceived cognitive workload for the parameters except for one—the self-directed group experienced a significantly higher task complexity compared to the variable practice.

By allowing the participants in the variable practice group to practice till proficiency, they probably managed to overcome the increased difficulty and the increased cognitive workload, which resulted in a higher quality of training. However, we did not provide a more difficult training curriculum for our participants like Ali et al. did [35], but simply an initially more difficult training context. These findings align with Bjork et al., who argue that intentionally creating difficulties during training leads to an enhanced learning experience [36].

Based on our findings, we suggest that training centers should consider using the variable practice approach for basic laparoscopic simulator training, as it has several benefits. It accelerates skills acquisition and leads to higher transfer and retention. It is also more time-efficient for the trainees and the instructor, as assistance is mainly needed at the beginning of training (Fig. 4). Furthermore, the implementation of variable practice comes with no additional costs and could easily be added as an automatic function in virtual reality simulators. We are currently in a dialog with simulator manufacturers to create a variable practice mode where different tasks are presented in random order and removed from the training program once the proficiency criteria have been met.

A strength of our study is a larger sample size compared with previous studies, e.g., Shewokis et al. also examined the effect of variable practice on the Lapsim® virtual reality simulator but only included 10 participants, 5 in each group. Their results were mixed and could, therefore, not demonstrate the statistically significant superiority of variable practice.

A limitation of our study is that we did not examine the transfer of skills to a clinical setting. Previous studies have already shown that skills obtained on a virtual reality laparoscopy simulator are transferable to a clinical setting [3, 4]. It would have been interesting to examine if there was any difference in skills transfer to a clinical setting after having practiced on a simulator using the variable practice approach in contrast to the self-directed approach. Additionally, we did not include the procedural task in the intervention; it would have been interesting to see how this would have impacted the training time needed to acquire proficiency for the variable practice group. However, it would have altered our study design, and we could not have used it as a transfer test. Finally, we used medical students instead of novice surgeons without surgical experience for our study. The training curriculum was intended for new residents who are surgical novices but including residents in the study could possibly have resulted in a high drop-out rate due to junior doctors’ amount of work hours. Furthermore, it is highly unlikely that the residents would have lasted the duration of the trial without having performed any surgeries, which was an exclusion criterion. In our opinion, medical students are comparable to junior doctors without any surgical experience, as both groups do not have any practical experience with surgery. Furthermore, the results of this study, for the control group, are comparable to those obtained by medical doctors without surgical experience in a simulated setting using the same training program [22]. However, this would be interesting to explore further.

## Conclusion

Variable practice is superior to conventional self-directed training for proficiency-based laparoscopy simulator training. It leads to faster skills acquisition, higher transfer to a procedural task, and higher retention of skills.

### Supplementary Information

Below is the link to the electronic supplementary material.

Supplementary file1 (DOCX 18 KB)

## Data Availability

Data are obtainable through the corresponding author upon reasonable request.
