# Supplementary material for: Variable practice is superior to self-directed training for laparoscopic simulator training: a randomized trial
Source: Surg Endosc. 2024 Feb 6;38(4):1902–11. doi: 10.1007/s00464-024-10688-z (PMC10978673; doi:10.1007/s00464-024-10688-z)
Supplement: Supplementary file 1 — Supplementary file1 (DOCX 18 KB) [file 464_2024_10688_MOESM1_ESM.docx]

Supplementary material: Proficiency settings for Basic Skills tasks and Procedural task

Basic skill 1: Grasping

| Parameter | Requirements for proficiency level |
| --- | --- |
| Left instrument time (s) | < 45 |
| Left instrument path length (m) | < 2 |
| Left instrument angular path (degrees) | < 300 |
| Right instrument time (s) | < 45 |
| Right instrument path length (m) | < 2 |
| Right instrument angular path (degrees) | < 300 |
| Tissue damage (frequency) | < 3 |
| Maximum damage (mm) | < 5 |

Basic skill 2: Lifting and grasping

| Parameter | Requirements for proficiency level |
| --- | --- |
| Total time (s) | < 120 |
| Left instrument misses (%) | < 60 |
| Left instrument path length (m) | < 3.2 |
| Left instrument angular path (degrees) | < 600 |
| Right instrument misses (%) | < 60 |
| Right instrument path length (m) | < 3.2 |
| Right instrument angular path (degrees) | < 600 |
| Tissue damage (frequency) | < 5 |
| Maximum damage (mm) | < 15 |
| Grasper collided with left box (frequency) | < 10 |
| Left box lifted (frequency) | < 15 |
| Grasper collided with right box (frequency) | < 10 |
| Right box lifted (frequency) | < 15 |

Basic skills 3: Fine dissection

| Parameter | Requirements for proficiency level |
| --- | --- |
| Total time (s) | < 150 |
| Ripped or burned blood vessels | < 0 |
| Energy damaged on blood vessels (%) | < 20 |
| Ripped small vessels (%) | < 25 |
| Burned small vessels (%) | < 25 |
| Grasper path length (m) | < 0.5 |
| Grasper angular path (degrees) | < 120 |
| Grasper outside view (frequency) | < 2 |
| Grasper outside view (s) | < 4 |
| Cutter path length (m) | < 0.8 |
| Cutter angular path (degrees) | < 200 |
| Cutter outside view (frequency) | < 2 |
| Cutter outside view (s) | < 4 |

Basic skill 4: Cutting

| Parameter | Requirement for proficiency level |
| --- | --- |
| Total time (s) | < 200 |
| Rip failure (%) | < 25 |
| Drop failure (%) | < 25 |
| Cutter path length (m) | < 2 |
| Cutter angular path (degrees) | < 400 |
| Grasper path length (m) | < 1.8 |
| Grasper angular path (degrees) | < 400 |
| Max stretch damage (%) | < 100 |
| Tissue damage (frequency) | < 10 |
| Maximum damage (mm) | < 25 |

Procedural task: Ectopic Pregnancy

Parameters for the procedure: salpingectomy on the Lapsim® virtual reality simulator. *To reach the proficiency level, all the proficiency level requirements must be fulfilled by using the correct operation technique.*

| Parameters | Requirements for proficiency level |
| --- | --- |
| Total time (s) | < 280 |
| Left instrument path length (m) | < 2 |
| Left instrument angular path (degrees) | < 350 |
| Right instrument path length (m) | < 3 |
| Right instrument angular path (degrees) | < 450 |
| Blood loss (ml) | < 180 |
| Pool of blood (ml) | < 10 |
| Ovary Diathermy damage (s) | < 3 |
| Tube Cut:  Uterus distance (mm) | < 4 |
| Bleeding vessel cut (Yes/No) | No |
